# Concurrent Presentation of Pure Red Cell Aplasia and Myeloproliferative Neoplasm, Unclassifiable With JAK2 and MPL Mutations

**DOI:** 10.1155/crh/9728317

**Published:** 2025-12-12

**Authors:** Qiuyang Li, Lin Tan, Xuejiao Wang, Qiwei Fan

**Affiliations:** ^1^ Department of Hematology, The People’s Hospital of Dali Bai Autonomous Prefecture, Dali, Yunnan Province, China; ^2^ Department of Hematology, The First Affiliated Hospital of Kunming Medical University, Kunming, China, kmmc.cn; ^3^ Department of Pneumology, The People’s Hospital of Chuxiong City, Chuxiong City, Yunnan Province, China

**Keywords:** JAK2 V617F, MPL W515, myeloproliferative neoplasm, pure red cell aplasia, ruxolitinib, unclassifiable (MPN-U)

## Abstract

Pure red cell aplasia (PRCA) is a rare hematologic disorder characterized by normocytic anemia and severe reticulocytopenia. The co‐occurrence of PRCA and myeloproliferative neoplasm (MPN) with JAK2 and MPL mutations is exceptionally rare. This case involves a patient who initially presented with anemia and thrombocytosis. Following a diagnosis of PRCA, the treatment with immunosuppressive therapy effectively increased her hemoglobin levels. Genetic testing revealed the presence of JAK2 V617F and MPL W515L mutations. The bone marrow biopsy results indicated MPN‐U, followed by a subsequent biopsy revealing myelofibrosis secondary to MPN‐U. Subsequently, ruxolitinib was administered. This case highlights the significance of pathological examination and genetic mutation testing in achieving precise differential diagnoses in MPNs. Additionally, it demonstrates effective management strategies for patients diagnosed with PRCA and MPN with JAK2 and MPL mutations. The use of ruxolitinib and cyclosporin A has been shown to be beneficial for such patients. And the use of ruxolitinib decreases the dosage of cyclosporin A, indicating that ruxolitinib may have a therapeutic effect on PRCA.

## 1. Introduction

Pure red cell aplasia (PRCA) is a normocytic normochromic anemia characterized by a significant reduction or absence of erythroid precursors, while maintaining normal production of megakaryocyte and white cell precursors. PRCA is frequently associated with conditions such as thymoma, autoimmune disease, infections (particularly human parvovirus B19) [1], lymphoproliferative disorders, myeloproliferative diseases, hematopoietic stem cell transplantation, and the use of certain medications [2–4]. Myeloproliferative neoplasm unclassifiable (MPN‐U) demonstrates the features of an MPN, while failing to meet the diagnostic criteria for an alternative specific MPN as defined by WHO criteria [5]. The coexistence of PRCA and MPN‐U in a single patient has not been reported before. Additionally, MPN with mutations in Janus Kinase 2 (JAK2) and MPL represents an uncommon condition with limited documentation in the existing literature [6–10]. In this report, we present the case of a 85‐year‐old patient diagnosed with PRCA and MPN‐U with JAK2 V617F and MPL W515L mutations.

## 2. Case Presentation

On March 26th, 2023, an 85‐year‐old female patient presented with a complaint of progressive fatigue lasting over one year. She had previously been diagnosed with severe anemia at a local hospital and had received treatment with iron supplements, which proved ineffective. Prior to her admission to our hospital, her condition worsened, manifesting as severe fatigue, dizziness, cough, dyspnea, and a sudden syncope with fecal incontinence. Upon admission, laboratory tests revealed macrocytic anemia with a hemoglobin level of 39 g/L. The patient presented with a reticulocyte count of 0.05 × 10^12^/L, a platelet count of 456 × 10^9^/L, and a WBC count of 9.32 × 10^9^/L. The initial peripheral blood smear showed no evidence of teardrop cells, immature granulocytes, or nucleated erythrocytes. Computed tomography of the chest revealed inflammatory opacities in both lungs, pleural effusion, and a space‐occupying lesion in the anterior mediastinum. The physical examination did not reveal splenomegaly. The syncope was determined to be caused by cerebral anoxia resulting from severe anemia and respiratory failure. The patient received emergency transfusion, oxygen therapy, and anti‐infection therapy. Further investigation into the cause of the anemia included testing for vitamin B12, folic acid, and ferritin levels, which did not indicate any evidence of nutritional deficiencies. The bone marrow smear exhibited an active proliferation of granulocytes and megakaryocytes, along with a decreased erythropoiesis, resulting in a myeloid/erythroid ratio of 49.33:1. Erythroblasts were notably absent. And there was no indication of hemolysis. The antinuclear antibody (ANA) test revealed a positive result for ANA, and negative for other antibodies. The lactic dehydrogenase level was measured at 399 IU/L. These findings collectively suggest a suspected diagnosis of PRCA, with thrombocytosis likely attributed to infection. The patient experienced symptomatic relief following transfusion, oxygen therapy, and anti‐infection therapy, subsequently leading to discharge.

On May 5th, 2023, she was admitted again due to increased fatigue. Blood count analysis revealed normochromic normocytic anemia, with a hemoglobin level of 34 g/L. The white blood cell count was within normal limits, the platelet count was 450 × 10^9^/L, and the reticulocyte count was 0.003 × 10^12^/L. Further diagnostic tests were conducted to confirm the diagnosis of PRCA and identify the underlying cause. A bone marrow biopsy demonstrated active bone marrow proliferation (40%) and decreased erythroid proliferation, with minimal reticulin or collagen fibrosis (Grade 1). The flow cytometry analysis indicated the absence of lymphocytes displaying abnormal immunophenotype, thereby ruling out the possibility of large granular cell leukemia. The contrast‐enhanced computed tomography of the thymus identified a potentially enlarged lymph node in the thymic region, which appears benign on contrast CT. Given the patient’s advanced age and potential inability to tolerate surgery, a biopsy of the mediastinal mass was not performed. The patient received a diagnosis of PRCA and was initiated on a treatment regimen consisting of 40 mg prednisone daily and 100 mg cyclosporin A twice daily. The patient’s infectious symptoms (including cough) have improved. Follow‐up contrast‐enhanced chest CT demonstrates a resolving pulmonary infection compared to prior imaging. Persistent thrombocytosis raises clinical suspicion for MPN. While current bone marrow biopsy is nondiagnostic for classical MPN subtypes, close monitoring of platelet counts during therapeutic follow‐up is warranted.

Following her discharge from the hospital, the prednisone was tapered gradually. Concurrently, an increase in hemoglobin levels was observed, accompanied by a mild renal injury. So the dosage of cyclosporin A was gradually adjusted to 25 mg twice daily.

**Figure 1:**
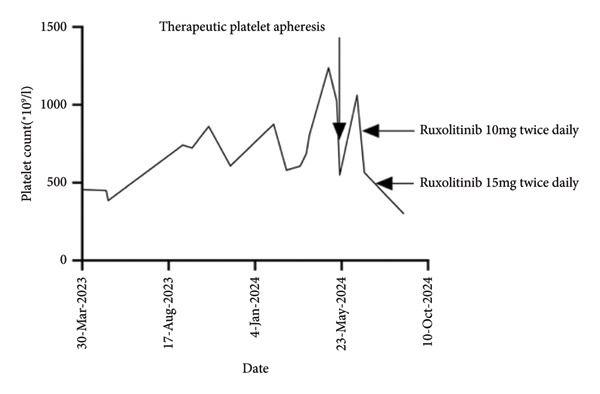
Graph of the platelet count from March 30th, 2023, to September 1st, 2024.

On March 26th, 2024, she presented with symptoms of fatigue and dizziness. Throughout the period spanning from March 2023 to March 2024, the platelet count exceeded 450 × 10^9^/L most of the time (see Figure 1). In light of these findings, a bone marrow biopsy and next‐generation sequencing (NGS) panel were conducted to assess MPNs. The bone marrow biopsy findings indicate hypercellular marrow (75%) with decreased erythroid proliferation and minimal fibrosis (Grade 1). Atypical megakaryocytes were not present, with 3–7 megakaryocytes observed per high power field. NGS revealed pathogenic variants including JAK2 V617F (variant allele frequency [VAF] 0.45%) and MPL W515L (VAF 34.5%). Concurrently, the hemoglobin level decreased to 83 g/L. Considering anemia may be the reason for fatigue and dizzy, the dosage of cyclosporin A was modified to 75 mg twice daily. The constellation of peripheral blood indices, bone marrow histopathology, and molecular genetics (NGS) supports diagnosing the patient with MPN‐U and PRCA at this clinical juncture. Due to the significant improvement in the patient’s anemia following cyclosporine and prednisone administration, combined with the bone marrow morphology findings, we inferred that her anemia was caused by PRCA rather than by the MPN.

Additionally, aspirin was prescribed at a daily dose of 100 mg. Subsequently, the dosage of cyclosporin A was further adjusted to 100 mg twice daily due to inadequate control of the anemia.

On May 5th, 2024, the patient presented at our hospital once again with symptoms of recurring fatigue and dizziness. Laboratory results revealed a hemoglobin level of 110 g/L, a platelet count of 1024 × 10^9^/L, and a WBC count of 8.57 × 10^9^/L. The follow‐up peripheral blood smear revealed teardrop cells, immature granulocytes, and nucleated erythrocytes. Subsequent bone marrow biopsy indicated hypercellularity of myeloid cells and megakaryocytes (75%), along with increased fibrosis (fibrosis Grade 2) and atypical megakaryocytes. The patient was ultimately diagnosed with PRCA and myelofibrosis secondary to MPN‐U. Genetic testing revealed the presence of JAK2 V716F and MPL W515L mutations, with the VAF not specified. At that time, the patient’s lactate dehydrogenase (LDH) level was 314 U/L, but physical examination revealed no splenomegaly. Due to the patient’s significant fatigue, she received therapeutic platelet apheresis, and the treatment with ruxolitinib was initiated at a dosage of 5 mg twice daily. Ruxolitinib may induce anemia in certain patients. Considering the presence of underlying PRCA in this case, dose titration was conducted with caution during the initiation of ruxolitinib therapy. During the outpatient follow‐up period, the dosage of cyclosporin A was modified to 75 mg twice daily in response to the patient’s hemoglobin level. Due to a rising platelet count (1060 × 10^9^/L) and persistent fatigue, the ruxolitinib dose was increased to 10 mg twice daily during subsequent treatment. On June 29th, 2024, the patient’s blood count revealed a hemoglobin level of 120 g/L, a WBC count of 9.76 × 10^9^/L, and a platelet count of 566 × 10^9^/L, indicating the efficacy of ruxolitinib. At the same time, the patient reported significant alleviation of fatigue. Additionally, the patient’s creatinine level remained stable.

In August 2024, the dosage of ruxolitinib was modified to 15 mg twice daily, and the dosage of cyclosporin A was adjusted to 50 mg twice daily. Despite the adjustment, her hemoglobin level did not decrease like before. In September 1st, 2024, the blood count revealed a hemoglobin level of 121 g/L, a WBC count of 8.50 × 10^9^/L, and a platelet count of 299 × 10^9^/L.

## 3. Discussion

Changes in the bone marrow biopsy from MPN‐U to myelofibrosis secondary to MPN‐U were observed in this patient. The diagnosis of MPN‐U in this patient requires differentiation from pre‐PMF and ET. All three conditions can present with significant thrombocytosis accompanied by Grade 1 or Grade 0 fibroses. However, according to the WHO criteria, both pre‐PMF and ET exhibit atypical megakaryocytes on bone marrow biopsy—characterized by “cloud‐like” nuclei in the former and “stag‐horn‐like” nuclei in the latter [11, 12]. She should have been diagnosed with MPN‐U in March 2024, not pre‐PMF or ET, as her bone marrow biopsy showed no megakaryocyte atypia, which does not meet the diagnostic criteria for pre‐PMF or ET. However, her platelet count remained above 450 × 10^9^/L for more than 3 months, she carried JAK2 and MPL gene mutations, and she did not meet the diagnostic criteria for other MPNs—a combination fulfilling the diagnostic criteria for MPN‐U [13]. Given that the detection of atypical megakaryocytes in bone marrow biopsies may be influenced by the pathologist’s experience or the quality of bone marrow sampling, and given the presence of atypical megakaryocytes in the patient’s most recent bone marrow biopsy, the possibility of prior diagnoses of ET or pre‐PMF cannot be ruled out. Only a limited number of cases have demonstrated concurrent JAK2 and MPL mutations, which have been associated with myelodysplastic/MPNs, polycythemia vera, and essential thrombocythemia [6–8, 10]. Additionally, a case has been reported with primary myelofibrosis and triple mutations in JAK2, CALR, and MPL [9]. Cases of MPNs with double or triple mutations do not appear to have clear clinical relevance based on the limited current evidence, and treatment approaches for these patients vary.

The JAK‐STAT signaling pathway plays a crucial role in the activity of various cytokine molecules and is essential for both adaptive and innate immunity, as well as hematopoiesis. This pathway also enhances the effects of several inflammatory cytokines implicated in the pathogenesis of autoimmune conditions such as ankylosing spondylitis, psoriasis, rheumatoid arthritis, and inflammatory bowel disease [14]. Moreover, in patients with myelofibrosis, activation of JAK‐STAT signaling influences the differentiation of T lymphocytes and dendritic cells [15]. The development of myelofibrosis is thought to be associated with the activation of JAK‐STAT signaling. Mutations in JAK2, MPL, and CALR genes can result in activation of this signaling pathway [16]. Acquired PRCA is often regarded as an autoimmune disease. In most cases, the pathophysiology of PRCA is linked to autoimmune mechanisms, including humoral immunity—where inhibitors of erythropoiesis have been demonstrated—and T‐cell–mediated processes. The T‐cell–mediated autoimmune process is considered the primary pathophysiological mechanism underlying acquired PRCA, explaining the efficacy of cyclosporine A as a treatment for this condition. PRCA can be idiopathic or secondary to conditions such as autoimmune disorders, infections, medications, and malignancies [3]. In this patient, since an MPN diagnosis was not initially established, it could not be definitively determined whether her PRCA was secondary to the MPN or occurred independently.

It is noteworthy that a distinct form of myelofibrosis, known as autoimmune myelofibrosis (AIMF), exists. AIMF often occurs in the setting of underlying autoimmune diseases. Furthermore, among reported AIMF cases, JAK2, CALR, and MPL gene mutations are almost invariably negative. Another characteristic feature of AIMF is its excellent response to immunosuppressive therapy [17]; the vast majority of AIMF patients achieve histopathological improvement in the bone marrow with such treatment [18]. In this patient, JAK2 and MPL gene mutations were positive. Therefore, she should not be diagnosed with AIMF, but rather with myelofibrosis secondary to MPN‐U. Concurrently, this patient should be closely monitored with bone marrow biopsies to assess morphologic changes induced by cyclosporine and ruxolitinib.

Ruxolitinib is a JAK inhibitor used in the treatment of MPNs [19]. Inhibitors of the JAK‐STAT signaling pathway, such as ruxolitinib, have also been used in the treatment of certain autoimmune diseases. In this case, ruxolitinib was beneficial: It reduced the required dose of cyclosporine while maintaining hemoglobin levels above 120 g/L, and it also lowered the platelet count. Further clinical investigation is warranted to determine whether ruxolitinib could be effectively used in the treatment of PRCA.

## Ethics Statement

This case report does not need any ethical approval.

## Consent

The patient has provided informed consent for publication of this case.

## Disclosure

The authors have no affiliations with or involvement in any organization or entity with any financial interest in the subject matter or materials discussed in this manuscript.

## Conflicts of Interest

The authors declare no conflicts of interest.

## Author Contributions

Qiuyang Li: conceptualization, data curation, original draft, and writing. Lin Tan: review and editing. Xuejiao Wang: data collection. Qiwei Fan: software.

## Funding

This research received no specific grant from any funding agency in the public, commercial, or not‐for‐profit sectors.

## Data Availability

The data that support the findings of this study are available from the corresponding author upon reasonable request. The data are not publicly available due to privacy or ethical restrictions.
